# The Medical Digest, or Busy Practitioner's Vade-Mecum

**Published:** 1891-09

**Authors:** 


*The Medical Digest, or Busy Practitioner's Vade-Mecum.* Being a Means of Readily Acquiring Information upon the Principal Contributions to Medical Science during the last Fifty Years. By RICHARD NEALE, M.D. Lond. Third Edition. Pp. xi.—794—cxxxii. London: LEDGER, SMITH & CO. 1891.

The third edition of this important work testifies to the unconquerable industry of Dr. Neale. Although the value of the book is somewhat minimised by the limited number of journals with which it deals, it is nevertheless one that should be in every public library and within reach of every diligent student of the medical sciences. When reviewing the second edition and its 1886 appendix, we pointed out that the work is much more than a bare classified reference to papers, and we should again like to emphasise this; for, owing to Dr. Neale's system, a needless search is not seldom obviated, as the leading thought of the communication is often given.

It would be unreasonable to expect that in a work of this kind there should be no errors of arrangement. Recently we had occasion to look up the references to the operation of uniting the sides of an anal fistula. In Section 889. 5, after the name of Reeves, the mode of printing makes it appear that the three references are to papers by this surgeon. The second reference is wrong, and the third should have Stephen

Smith's name prefixed. But this is a tiny spot on a sun. We owe Dr. Neale an immense debt of gratitude for affording us this real help, and for absolving our nation from the charge of bibliographical indolence which might otherwise be fairly brought against it.

